# CATANA: an online modelling environment for proteins and nucleic acid nanostructures

**DOI:** 10.1093/nar/gkac350

**Published:** 2022-05-11

**Authors:** David Kuťák, Lucas Melo, Fabian Schroeder, Zoe Jelic-Matošević, Natalie Mutter, Branimir Bertoša, Ivan Barišić

**Affiliations:** Molecular Diagnostics, AIT Austrian Institute of Technology, 1210 Vienna, Austria; Eko Refugium, 47240 Slunj, Croatia; Visitlab, Faculty of Informatics, Masaryk University, Brno 602 00, Czech Republic; Molecular Diagnostics, AIT Austrian Institute of Technology, 1210 Vienna, Austria; Eko Refugium, 47240 Slunj, Croatia; Molecular Diagnostics, AIT Austrian Institute of Technology, 1210 Vienna, Austria; Eko Refugium, 47240 Slunj, Croatia; Department of Chemistry, Faculty of Science, University of Zagreb, Horvatovac 102a, HR-10000 Zagreb, Croatia; Molecular Diagnostics, AIT Austrian Institute of Technology, 1210 Vienna, Austria; Department of Chemistry, Faculty of Science, University of Zagreb, Horvatovac 102a, HR-10000 Zagreb, Croatia; Molecular Diagnostics, AIT Austrian Institute of Technology, 1210 Vienna, Austria; Eko Refugium, 47240 Slunj, Croatia

## Abstract

In the last decade, significant advances have been made towards the rational design of proteins, DNA, and other organic nanostructures. The emerging possibility to precisely engineer molecular structures resulted in a wide range of new applications in fields such as biotechnology or medicine. The complexity and size of the artificial molecular systems as well as the number of interactions are greatly increasing and are manifesting the need for computational design support. In addition, a new generation of AI-based structure prediction tools provides researchers with completely new possibilities to generate recombinant proteins and functionalized DNA nanostructures. In this study, we present Catana, a web-based modelling environment suited for proteins and DNA nanostructures. User-friendly features were developed to create and modify recombinant fusion proteins, predict protein structures based on the amino acid sequence, and manipulate DNA origami structures. Moreover, Catana was jointly developed with the novel Unified Nanotechnology Format (UNF). Therefore, it employs a state-of-the-art coarse-grained data model, that is compatible with other established and upcoming applications. A particular focus was put on an effortless data export to allow even inexperienced users to perform *in silico* evaluations of their designs by means of molecular dynamics simulations. Catana is freely available at http://catana.ait.ac.at/.

## Graphical Abstract

**Graphical Abstract:**
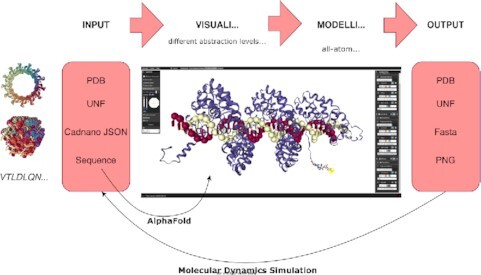
Modelling workflow of Catana.

## INTRODUCTION

Novel methods in the field of bio-nanoengineering allow to precisely design proteins (1) and DNA nanostructures (2) leading to a wide range of new applications in medicine, biotechnology and engineering. To investigate protein–protein interactions directly in cells, Martel *et al.* engineered a split horseradish peroxidase (HRP) comprising two non-catalytic subunits (3). Each subunit was fused to two specific proteins of interest. If the two proteins interacted, the HRP subunits came into close proximity, refolded, and reassembled a catalytically active HRP-fusion protein complex that was exploited for the visualization of the protein–protein interactions. Another bio-nanoengineering example illustrating the high potential of functionalized DNA nanostructures in medicine is a DNA-based nanorobot that was injected into mice as a cancer therapeutic (4). The thrombin-functionalized nanostructure was able to specifically bind to surface proteins of tumour cells via aptamers and to perform a structural change upon target recognition resulting in the activation of thrombin proteins that led to the destruction of the tumour. These two highlighted studies are representative examples in the rapidly advancing nanoengineering field and illustrate the emerging possibilities for novel *in vitro* and *in vivo* applications.

Computational tools are essential for the design of such nanostructures and can significantly facilitate their functionalization and *in silico* evaluation, thus helping to reduce time- and resource-intensive wet lab experiments. While it remains extremely challenging to identify design paradigms for proteins, the base complementary of nucleic acids allows the creation of computational algorithms for the predictable design of DNA nanostructures. The release of Cadnano democratized the DNA nanotechnology field giving every user the possibility to design self-assembling DNA nanostructures resulting in a remarkable number of new studies (5). A manifold of other DNA nanostructure design tools was subsequently published, providing additional design features to the users (Daedalus (6); Adenita (7); Magic DNA (8); V-helix (9); Tiamat (10); Vivern (11); Oxview (12)). However, most of these design tools were either dependent on commercial software solutions and platforms, or not fully functional, especially with respect to the 3D visualization of the nanostructures and free-hand manipulations. In most cases, the tools and the underlying data models also did not allow for an *in silico* evaluation of the design using molecular dynamics simulations and were limited to nucleic acids only. In contrast to protein-based structures, however, it was nevertheless possible to generate DNA nanostructures with arbitrary shapes using these tools.

A shortcoming of pure DNA-based nanostructures concerning potential applications is that DNA is in principle catalytically not active. As an exception to this rule, a few DNAzymes such as HRP-mimicking G-quadruplexes have been reported, but their catalytic activity is orders of magnitude lower compared to their protein-based counterparts (13,14). RNA-based catalysts have also been reported, but their biological instability is a significant challenge for a wide range of applications (15). Thus, proteins remain the molecules of choice to perform enzymatic reactions, to interact with other molecules, and to functionalize nanostructures. The main challenge concerning the exploitation of proteins for biotechnological applications is that their folding behaviour is poorly understood. A breakthrough in this field was recently achieved by applying artificial intelligence (AI)-based approaches to address this problem (16,17). Refining and applying these structure prediction tools will allow us, in the future, to rationally design proteins and enzymes, to precisely develop recombinant and fusion proteins, and to functionalize nanostructures.

In this work, we present the online modelling environment Catana for the creation of recombinant proteins and the functionalization of nucleic acid nanostructures. Catana provides a user-friendly interface with novel editing functionalities for performing a multitude of tasks, which otherwise require several different tools (VMD, Chimera, Maestro, etc.). It is especially useful for the creation of large macromolecules or macromolecular complexes, such as protein–protein, protein–DNA, DNA origami complexes, and fusion proteins. In addition, we integrated the AI-based tool AlphaFold (17) to predict the tertiary structure of proteins based on their amino acid sequence, which is especially useful when 3D structures are not available, or their structural files contain large portions of missing residues. Further, Catana was developed with native support for the new nanotechnology file format UNF (18) to facilitate the transfer and conversion of nanostructure designs made in external tools such as Cadnano and oxView. Finally, Catana was optimized to create structural files for molecular dynamics simulations (MDS). To demonstrate the design possibilities of Catana, we created two recombinant fusion proteins and functionalized a DNA nanostructure with protein pores.

## MATERIALS AND METHODS

### Overview of features

The main focus of Catana lies on the development of a pipeline for the design of recombinant proteins and functionalized nanostructures. However, we ensured that the features and user interface of Catana are suitable also for other types of tasks and use cases, giving the user freedom in the workflow.

The overall feature set of Catana can be divided into several higher-level categories, as presented in the following sections.

### Visualization

Catana can visualize both all-atom and coarse-grained structures using different representations and colour schemes, allowing the users to perceive the data using different abstraction levels. Moreover, the visualized content can be dynamically filtered and coloured in order to emphasize certain structural parts. Thus, the visual side of the web application is highly customisable, facilitating the exploration, idea-conveying, and dissemination tasks. Using a computer with Nvidia GeForce GTX 1070 graphics card, Catana was verified to interactively render all-atom structures with >200 000 atoms and coarse-grained structures with >50 000 nucleotides.

### Modelling toolkit

Apart from the visualizations, Catana focuses on modelling of structures, either starting from scratch or using an existing structure as a basis. In any case, all the modelling happens in real-time, directly in the user's browser. The supported modelling operations can be divided into two main groups, based on the area of focus: all-atom modelling and coarse-grained modelling. In the first area, Catana allows to remove parts of a structure, mutate amino acid residues or append new ones to the polypeptide chain. As for the second group, core operations known from the DNA nanotechnology domain are supported – for example, creating of single and double strands from scratch, breaking of strands, connecting them into one, and setting their sequence.

### Structural assembly

The individual structures loaded into Catana can be independently visualized, modified and translated in space. To support the creation of superassemblies of structures, gizmos for translation and rotation were implemented, allowing to constrain the rotation or translation only along a single axis. To make the positioning of structures even easier, the users can also select the operation for translation or rotation and simply drag the structure along a camera-aligned plane. Finally, when the structures are positioned as desired, the user can export the whole assembly as one file.

### Data models conversion

To foster the interoperability between the all-atom and coarse-grained structures, and increase the usability of the outcomes for molecular dynamics simulations, Catana allows to convert between these two descriptions, respectively data models, directly via the user interface. The conversion from coarse-grained data model can generate up to 99 999 atoms and we are working on ways to go beyond this limit. The opposite conversion does not have a strict limit onto the size of the structure, as it is less challenging from the perspective of performance and export formats.

### Data import and export

Catana can import and export various data formats as described in the following sections. Moreover, the input files can be loaded from the user's device, downloaded from a given URL, or directly imported from the RCSB database (https://www.rcsb.org/) or the AlphaFold Protein Structure Database (https://alphafold.ebi.ac.uk/).

### Alpha fold integration

AlphaFold is an AI-based tool that can predict the structure of a protein given only its sequence and is considered the most accurate and most reliable protein prediction tool available to date (19). As such, it fits the pipeline proposed by Catana and is integrated into the platform, allowing the users to start AlphaFold predictions and retrieve their results directly via the web application interface.

### Layout-based user interface

To provide a better support for different use cases, the user interface of Catana is split into several layouts, revealing only parts of the interface relevant to the given task. This way, the user can focus only on features relevant to their scenario. Moreover, as some operations are easier to perform via text commands than through a graphical user interface, Catana also provides a command-line interface.

### Supporting features

Apart from the main features, additional techniques and operations were implemented to facilitate the work in Catana. For example, the possibility to duplicate structures, visualize their sequences, or superimpose them onto each other.

### Architecture

Catana is designed to be a web-based application, providing easy access to its users, irrespective of their operating system. For this reason, the core feature set of Catana is developed to be client-side. Only computationally expensive features, such as protein structure prediction, which are not feasible for client-side execution, are running on a dedicated server. We have implemented a microservice architecture consisting of a webserver hosting an API, a light-weight task manager based on Celery and containerized workers. This architecture allows simple maintenance and can easily be extended to other heavy tasks. From the perspective of the user, all of the features can be accessed in a unified manner via the Catana user interface, providing a simple workflow.

### Input and output

Catana is able to import files from a vast range of existing file formats, ensuring that many of the existing structures can be readily loaded into the application. The following list divides the supported means of input into three main categories and their most important file formats:

- All-atom structures: PDB, PDBx/mmCIF, SDF, MMTF
- Coarse-grained structures: UNF, Cadnano JSON
- General three-dimensional objects: OBJ, PLY

Since Catana focuses on modelling and modification of structures, available means of output also play an important role for the usability of the platform. Therefore, as of now, Catana can export data in four formats:

- *PDB file:* structures loaded into Catana can be further exported in a PDB format, potentially combining multiple source structures in one file.
- *UNF file:* thanks to this format, Catana can export whole molecular scenes, even those combining all-atom and coarse-grained structures in one file.
- *FASTA file:* Catana can export FASTA files storing sequences of the selected structures.
- *PNG file:* in order to allow generation of structural images and publication figures, Catana provides means of customisable screenshot generation.

### Data model

The data model is an integral part of the Catana application, as it influences various factors, ranging from performance, through visualizations, to compatibility with other software. Catana employs two core data hierarchies. The first one is focused on all-atom structures, describing their chains, residues and atoms, and is based on the NGL framework (20) data model. The second one is a coarse-grained data hierarchy which describes the individual structures up to the amino or nucleic acid level and employs a parametrization compatible with the UNF to facilitate the interoperability with other tools. The parametrization plays an important role in the case of atom generation, as it determines how precisely the position of atoms can be computed.

In the case of DNA, Catana employs a two-step generation procedure. First, the nucleotide atoms are placed based on the coordinates computed from the parameters. Then, for each nucleotide, various backbone conformations are compared, differing in their dihedral angles. The conformation achieving the optimal phosphodiester bond length is selected. To verify the suitability of the final models, MDS were performed comparing models of the DNA crystal structure with a Catana generated double helix (Supplementary Figures S1–S3).

### Implementation

Catana is built on top of the open-source NGL framework (20) and its web viewer, as this solution was designed for visualization of molecular structures and, therefore, offered a wide and verified feature set serving as a reasonable starting point. As a consequence, Catana is developed using some of the most common web technologies; namely TypeScript, WebGL, Three.js and Node.js. As for the server-side part, we used the Python language and frameworks built on top of it, such as Flask, to handle the communication between the frontend and backend parts of the codebase.

## RESULTS AND DISCUSSION

In order to showcase and verify some of the features of Catana, we present three use cases being also developed *in vitro* in our lab. They demonstrate how Catana can facilitate the design and assembly of various structures, all of it realized via an easily accessible web interface.

### Generation of fusion proteins

Colicin S4 is a bacteria-binding protein and was previously used for the detection of *Escherichia coli* in drinking water (21). In this work by Gutierrez del Rio *et al.*, GFP was fused with colicin to visualize the binding to *E. coli* cells.

**Figure 1.**
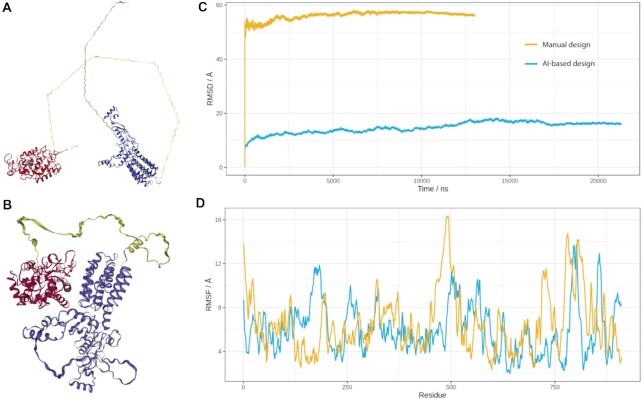
Illustrations of the designed fusion protein and their analysis using MDS. Screenshots of the colicin(blue)-HRP(red) fusion protein when (**A**) designed manually based on the structural PDB files and when (**B**) predicted by AlphaFold from the primary amino acid sequence. Filter-based colouring was used to highlight the different elements of the fusion protein. (C, D) Prior to MD simulations, systems were geometry optimized (1000 SD steps in vacuum followed by 3000 SD steps in explicit solvent), solvated and equilibrated. The (**C**) RMSD and (**D**) RMSF were calculated during the coarse-grained simulations. The RMSF was calculated for the first 13 μs of the simulation.

For our first Catana use case, we designed colicin-HRP fusion proteins to combine colicin with a more powerful reporter enzyme and, thus, to improve the sensitivity of the diagnostic assay. To minimize the effort of the *in vitro* experiments, we developed a design pipeline providing input for subsequent MDS analysis to verify the designs and their feasibility. To design this fusion protein, we opted for two approaches. Both resulting structures were later analysed using coarse-grained MDS (Figure 1C and Supplementary Figure S8).

The first approach relied on a manual design analogous to the standard procedure to create such fusion proteins. In this case, the user loaded two PDBs containing the source structures: 3FEW (https://www.rcsb.org/structure/3FEW) for colicin S4 and 1H5A (https://www.rcsb.org/structure/1H5A) for the HRP C1A.

Afterwards, the colicin structure was modified by manually appending the 65 amino acids missing in the original 3D structure of the PDB file. This was followed by appending a 100-amino-acid linker. Then, the user mutated selected residues of the HRP and added a his-tag. Finally, both proteins were positioned in close proximity to create the desired design, followed by exporting the final fusion protein as a PDB file. The new Catana features involved in adding long amino acid chains allowed us to significantly reduce the design ‘hands-on’ time compared to other *in silico* tools such as VMD or Maestro.

In the second approach, we used the amino acid sequence of the final fusion protein as an input for the AlphaFold structure prediction plugin to automatically create the structural model of the fusion protein. Both designs are illustrated in Figure 1. The manually-designed fusion protein is spread in space and requires a big simulation box comprising more water atoms resulting in simulation times increasing exponentially with the size of the box (Supplementary Figure S5A). The advantage of the AI-based approach is that the resulting structure is significantly more realistic and compact which massively facilitates a meaningful MDS analysis due to the smaller box and shorter simulation times (Supplementary Figure S5B). This example illustrates how the implemented AI-based structure prediction algorithms can support and improve the design of fusion proteins.

### Design of DNA–protein systems

Combining the advantages of DNA- and protein-based technologies opens up completely new possibilities for researchers and biotechnologists (22–24). To evaluate our data models and editors, we selected the plant transcription regulator protein TAL that specifically binds to a user-defined DNA sequence. We selected this protein because recombinant TAL fusion proteins were used in a pioneering work to fold DNA nanostructures into defined shapes illustrating the TAL’s high potential for future biotechnological applications (23).

First, we demonstrated with MDS that our DNA and protein data structure models are correct and that we could differentiate in the simulations TAL/DNA complexes with specific and random DNA, respectively (Supplementary Figures S4–S7). Second, the application of AlphaFold during the design process improved the precision of the design because the experimentally obtained TAL structure 3UGM (https://www.rcsb.org/structure/3UGM) lacked ca. 50 flanking amino acids that had to be added manually (Supplementary Figure S4).

**Figure 2.**
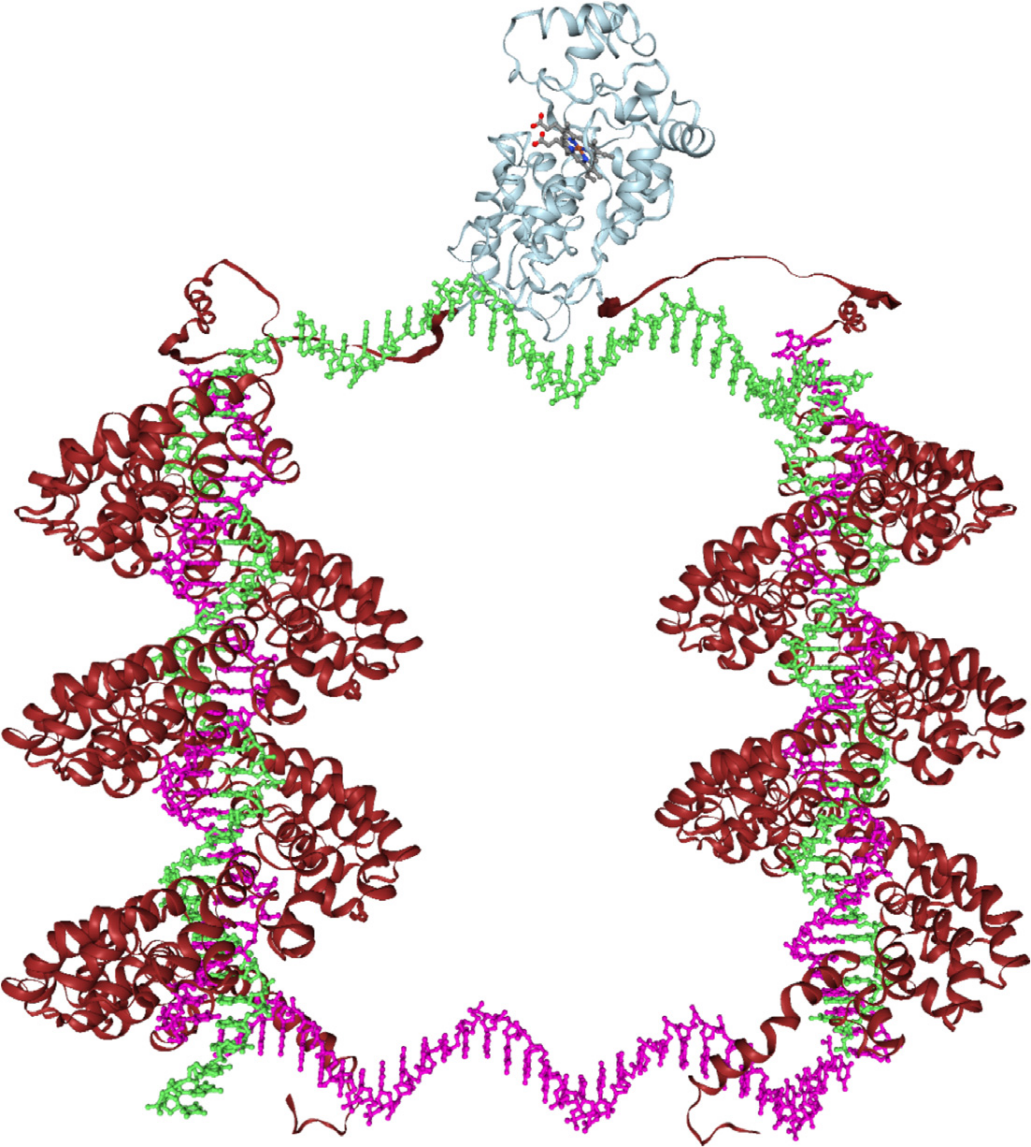
Screenshot of the split HRP-TAL fusion protein. Two TALs predicted by AlphaFold are connected to the N and C terminus of a split HRP while Catana-generated DNA acts as a clamp.

After confirming that our implemented data models were correct, we used Catana to design a recombinant fusion protein comprising two TAL proteins each flanked with a subunit of the split HRP developed by Martel *et al.* (Figure 2) (3). The user started the design process by requesting the AlphaFold plugin to predict the 3D structure of the TAL protein based on the primary amino acid sequence. Afterwards, the resulting PDB was loaded to Catana, where the user created a DNA double strand and positioned it accordingly to have the TAL wrapped around the DNA. After this step, the exported PDB was again imported to Catana, where the structure was duplicated, followed by loading the HRP isoenzyme 1CA (H5A PDB structure; https://www.rcsb.org/structure/1H5A). Upon transformation of the TAL complexes in close proximity to the HRP termini, minor modifications such as residue mutations, removal and re-appending, to prevent sterical clashes, were performed. Finally, a DNA clamp was added to bring the two HRP subunits to close proximity to allow refolding in the presence of the correct oligonucleotide. Due to the used data model, the resulting structure can be exported and analysed using both all-atom and coarse-grained MDS.

### Functionalization of DNA origami nanostructure

Functionalized DNA nanostructures hold a great potential for future theranostic applications. The DNA nanostructure acts as carrier protecting the load from the immune system and allows a target-specific release at the site of action (4). Furthermore, DNA nanostructures can be easily functionalized with aptamers and antibodies to bind to their targets and even induce structural switches releasing their cargo. The recent advances concerning the AI-based prediction of RNA structures indicate that the development of DNA aptamers might be also supported by AI-based algorithms in future (25). This would open up new possibilities for DNA nanostructure-based drugs in the field of personalized medicine.

**Figure 3.**
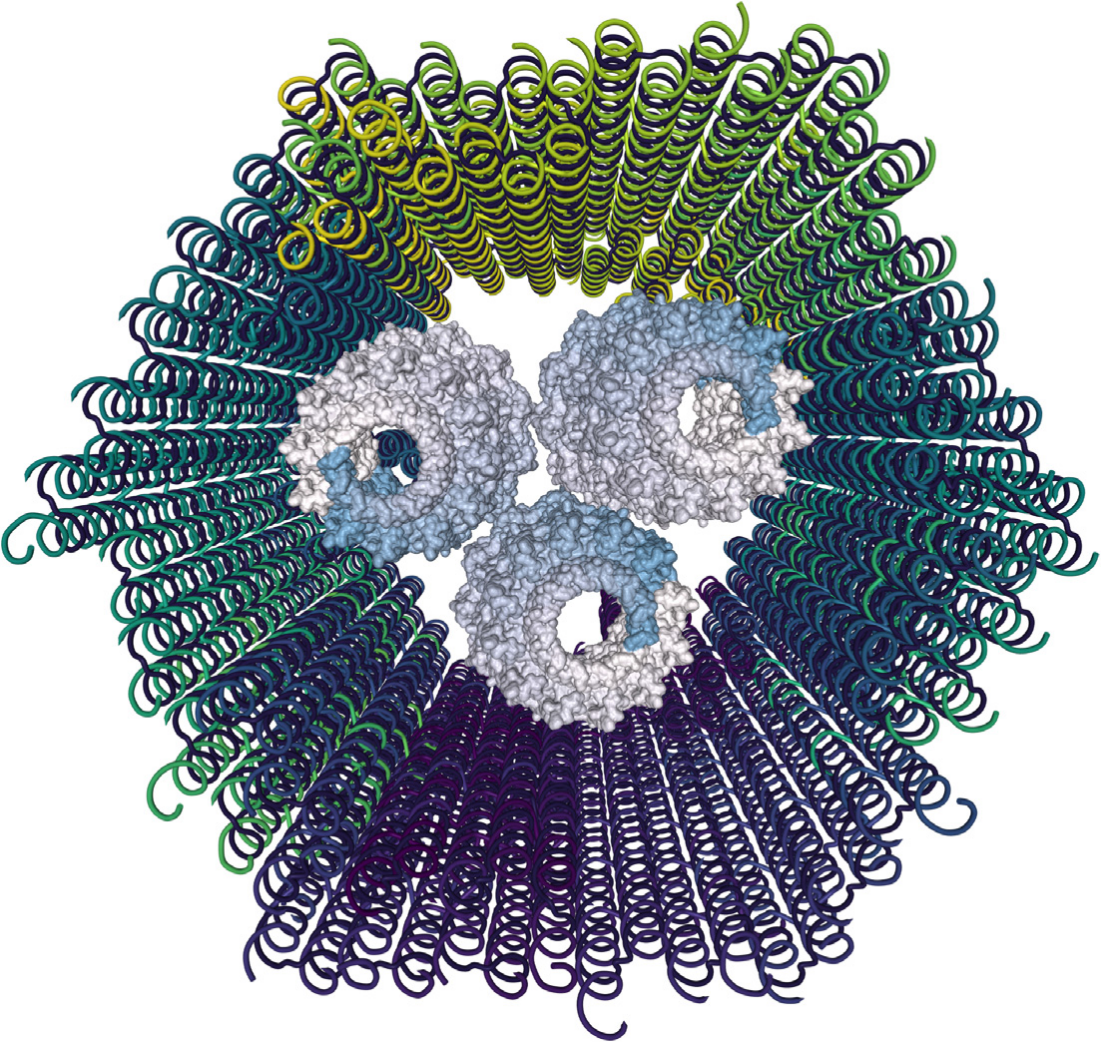
Screenshot of the design of the DNA origami carrier functionalized with three ClyA pores.

To demonstrate the complex design of such an example in Catana, we generated a two-component DNA nanocarrier functionalized with three protein pores Figure 3. The carrier is based on two DNA origami structures from the stator element of a DNA nanorotor (26), loaded as cadnano JSON files. Then, the ClyA pore (PDB 2WCD (https://www.rcsb.org/structure/2WCD) was loaded and duplicated multiple times. Afterwards, the user analysed the loaded structures and searched for optimal positions on the proteins and the DNA nanostructure to introduce linkers to covalently connect them. Due to the size of the object, coarse-grained simulations supporting protein–DNA systems such as ANM-oxDNA (27) are recommended for the structural evaluation.

## CONCLUSION

We have developed a user-friendly, web-based modelling tool to design and manipulate proteins, DNA and DNA nanostructures. State-of-the-art features from standalone tools for modelling, manipulation, and visualization of complex molecular systems were implemented in Catana. The AI-based AlphaFold algorithm was embedded to support the design of protein-based structures. Support for popular molecular modelling file formats was implemented to facilitate the exchange of designs between different software tools. The structural models were optimized to enable MDS analysis of the designs. Finally, we demonstrated Catana's features using three real use cases.

## Supplementary Material

gkac350_Supplemental_File
