# Supplementary material for: CATANA: an online modelling environment for proteins and nucleic acid nanostructures
Source: Nucleic Acids Res. 2022 May 11;50(W1):W152–8. doi: 10.1093/nar/gkac350 (PMC9252799; doi:10.1093/nar/gkac350)
Supplement: gkac350_Supplemental_File [file gkac350_supplemental_file.pdf]

Published online *Nucleic Acids Research, YYYY, Vol. xx, No. xx* **1–9**
doi:10.1093/nar/gkn000

SUPPLEMENTARY MATERIAL
CATANA: an online modelling environment for proteins and nucleic acid nanostructures

David Kuťák [1,2], Lucas Melo [1], Fabian Schroeder [1], Zoe Jelić-Matošević [3], Natalie Mutter [1], Branimir Bertoša [3] and Ivan Barišić [1]

[1] Business Unit Molecular Diagnostics, AIT Austrian Institute of Technology, 1210 Vienna, Austria [2] Visitlab, Faculty of Informatics, Masaryk University, Brno 602 00, Czech Republic [3] Department of Chemistry, Faculty of Science, University of Zagreb, Horvatovac 102a, HR-10000 Zagreb, Croatia

S1.1 *IN SILICO* EXPERIMENTS

The structures designed and assembled in Catana were evaluated using molecular dynamics simulations, in order to better understand their structural stability and behavior. The outcomes of these simulations are described in Figures S1 to S8.

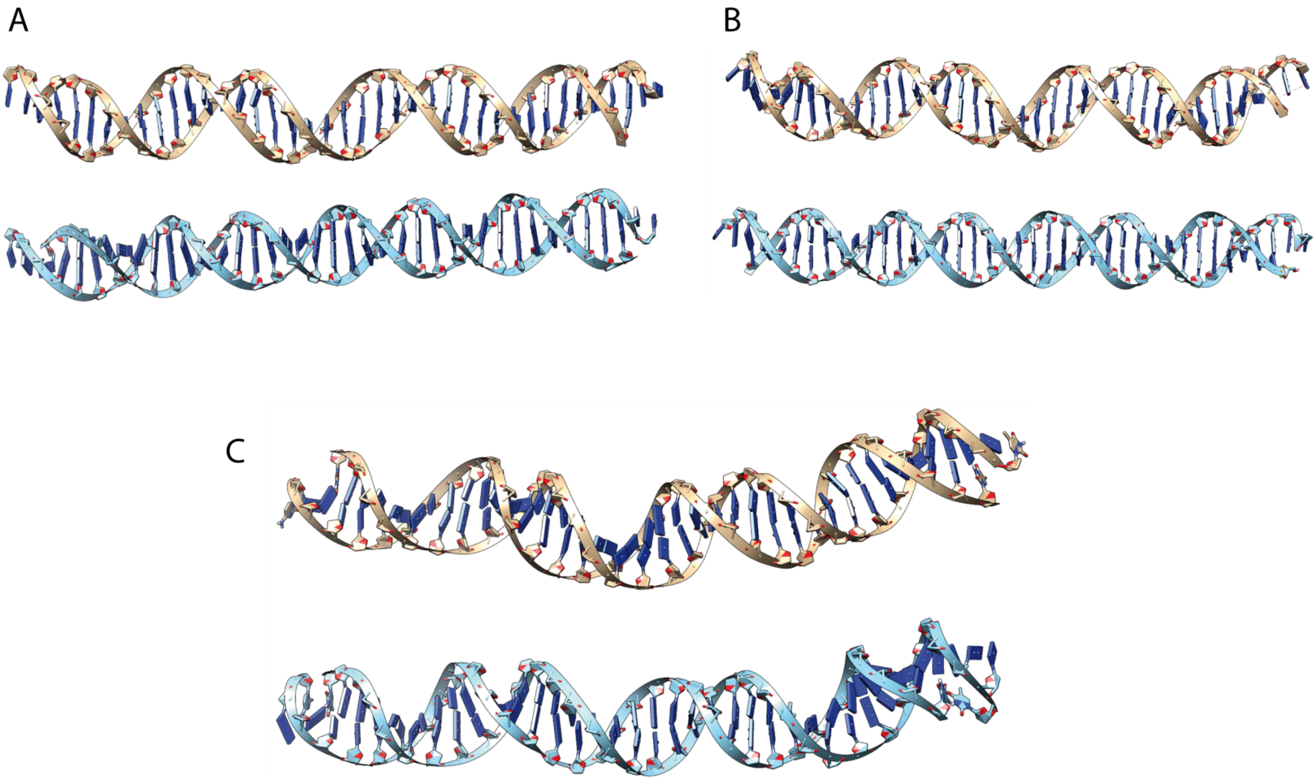

Figure S1. Screenshots of DNA structures prepared from the crystal structure (PDB ID: 3UGM) (top, yellow) and generated from scratch in Catana (bottom, blue). Both were subjected to MD simulations. (A) The starting structures, (B) the geometry optimised strctures (SD, 3000 steps), and (C) the structures after 10 ns of MD simulations in Amber20 (bsc1 force field) using explicit solvent model (TIP3) with PBC at 310 K (Langevin thermostat with the gamma value 3.0) are illustrated. Prior to the productions phase, the systems were equilibrated for 50 ps (NVT with temperature incensement, followed by 50 ps NTP).

© YYYY The Author(s)
This is an Open Access article distributed under the terms of the Creative Commons Attribution Non-Commercial License (http://creativecommons.org/licenses/by-nc/2.0/uk/) which permits unrestricted non-commercial use, distribution, and reproduction in any medium, provided the original work is properly cited.

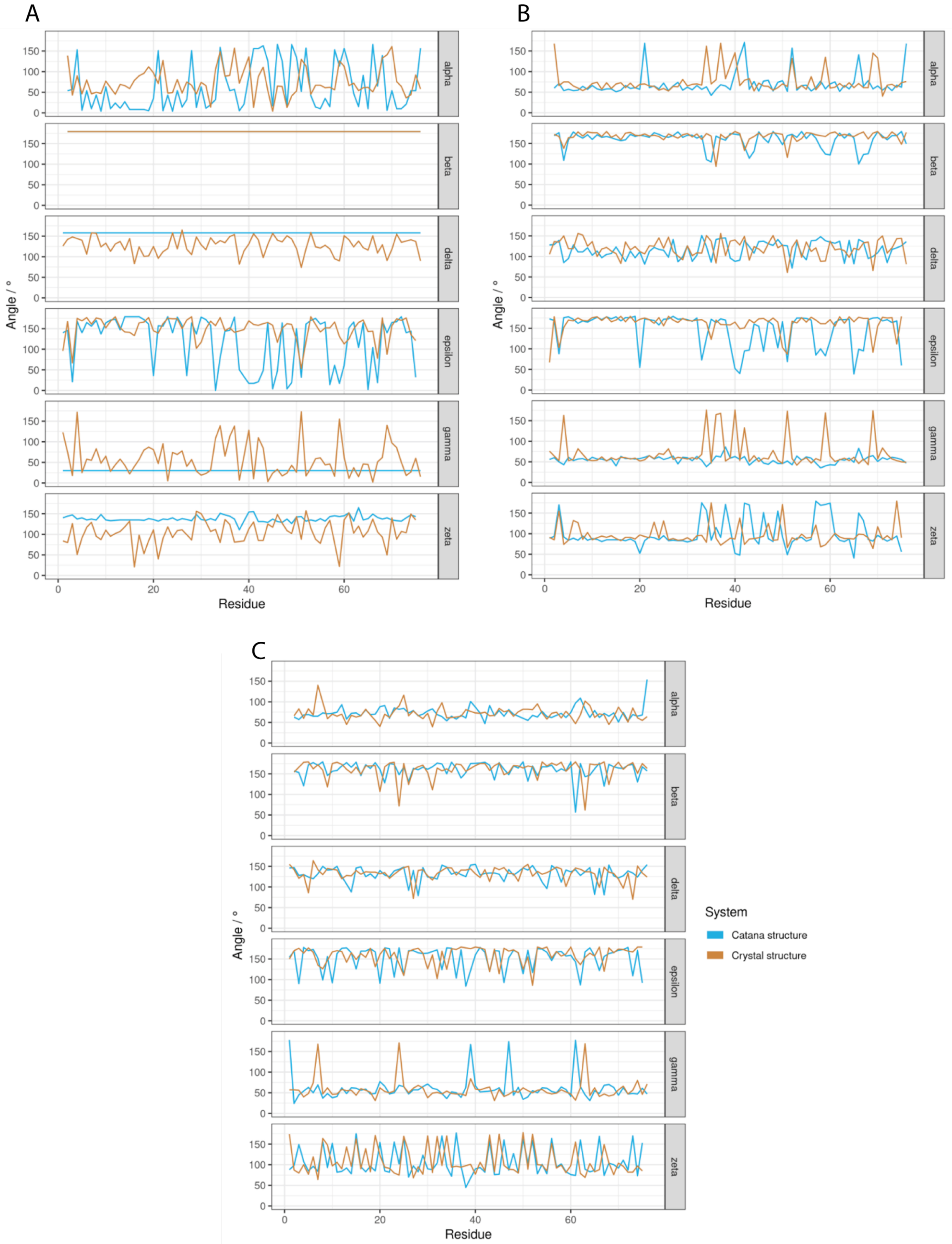

Figure S2. The DNA backbone dihedral angles of DNA structures obtained from the crystal structure (orange) and generated in Catana (blue). (A) The initial structures, (B) after geometry optimisation (SD, 3000 steps), and (C) after 10 ns of MD simulations in Amber20 (bsc1 force field) using explicit solvent model (TIP3) with PBC at 310 K (Langevin thermostat with the gamma value 3.0) are illustrated.

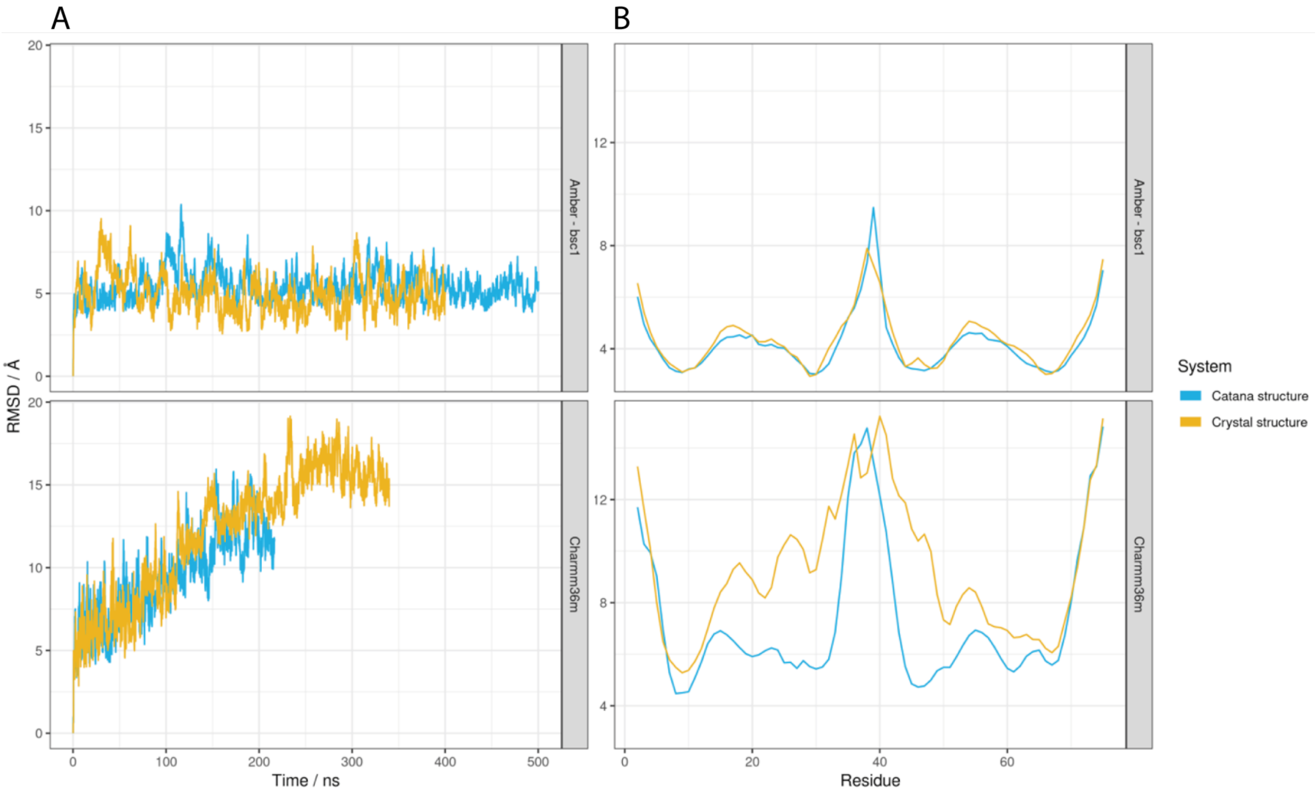

Figure S3. Comparison of (A) the changes in the RMSD of the DNA backbone and (B) the fluctuations of nucleotides (RMSF) during the MD simulations of the system prepared from the crystal structure (PDB ID: 3UGM) (yellow) and the one generated from scratch in Catana (blue). After energy minimisation (SD, 3000 steps) and 50 ps of equilibration (NVT with temperature incensement, followed by 50 ps NTP), the systems were subjected to production phase MD simulations in Amber20 (bsc1 force field) using an explicit solvent model (TIP3) with PBC at 310 K (Langevin thermostat with the gamma value 3.0).

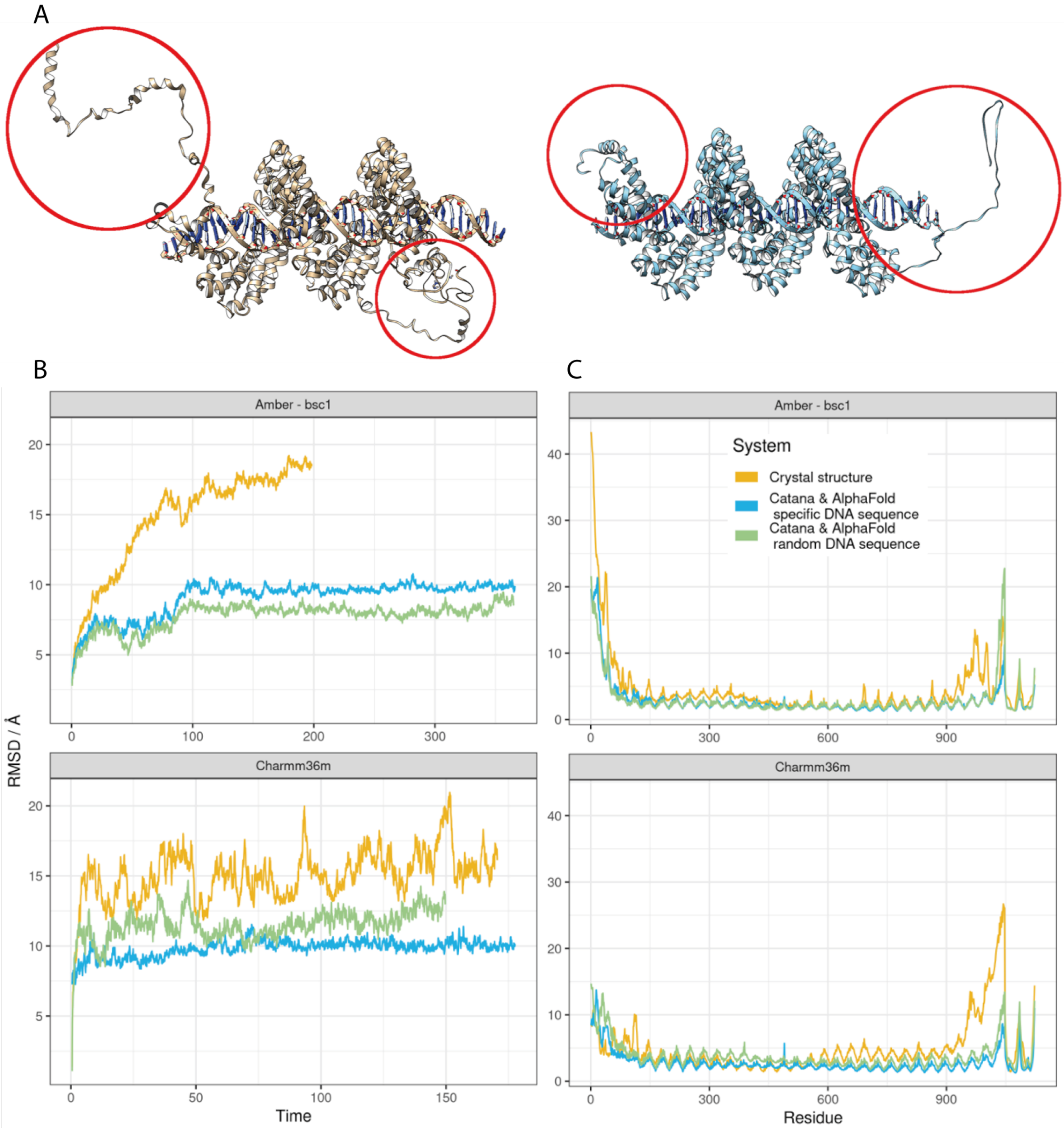

Figure S4. Evaluation of our data model for DNA-protein complex systems using the TAL protein in combination with DNA. After geometry optimisation (SD, 3000 steps) and equilibration with temperature incensement (NVT, followed by NTP), the systems were subjected to production phase at 310 K using the V-rescale algorithm (Gromacs, using Charm36m force field) or Langevin thermostat with the gamma value 3.0 (Amber20 using bsc1 force field). An explicit solvent model (TIP3) with PBC was used for the MD simulations. (A) The systems were modelled using two approaches: (left) the system was prepared starting from the crystal structure (PDB ID: 3UGM) and missing residues were added in CharmmGUI using the GalaxyFill algorithm (or alternatively in Chimera using MODELLER), (right) the system was prepared with Catana and the AlphaFold plugin. To analyse the specificity of the two DNA-protein interactions, we generated two AlphaFold-based structures - one with the same DNA sequence as in the crystal structure and another with a random DNA sequence (both DNA sequences were generated in Catana). (B) The stability of the systems was monitored by RMSD values of the residue center of mass of both the protein and DNA during the MD simulations. (C) Fluctuations of the systems were monitored by calculating RMSF of each residue during the MD simulations.

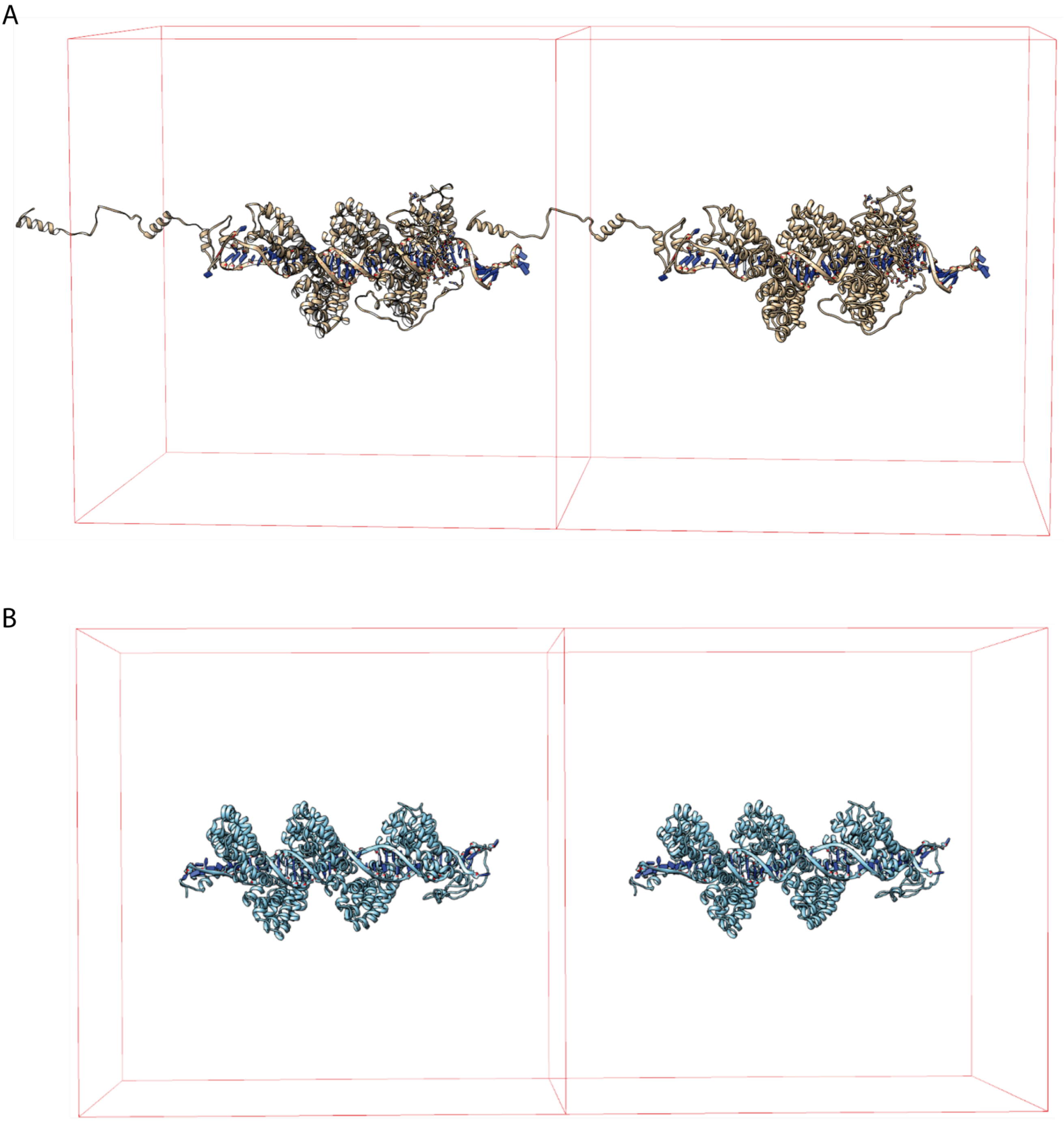

Figure S5. Screenshots illustrating the structures obtained from all-atom MD simulations of the TAL protein-DNA complex. After geometry optimisation (SD, 3000 steps) and equilibration with temperature incensement (NVT, followed by NTP), MD simulations were conducted at 310 K using the V-rescale algorithm (Gromacs, using Charm36m force field) or Langevin thermostat with the gamma value 3.0 (Amber20 using bsc1 force field). An explicit solvent model (TIP3) with PBC was used for the MD simulations. (A) The system prepared from the crystal structure (PDB ID: 3UGM) and missing residues added in CharmmGUI using the GalaxyFill algorithm (or alternatively in Chimera using MODELLER) and (B) the system prepared from Catana were analysed. (A) In the case of the manually prepared the system, the missing residues that were built using CharmmGUI "streched" out from the simulation box during simulation, crossing periodic boundary conditions and leading to self-interaction. The same did not happen with the molecular systems designed in Catana.

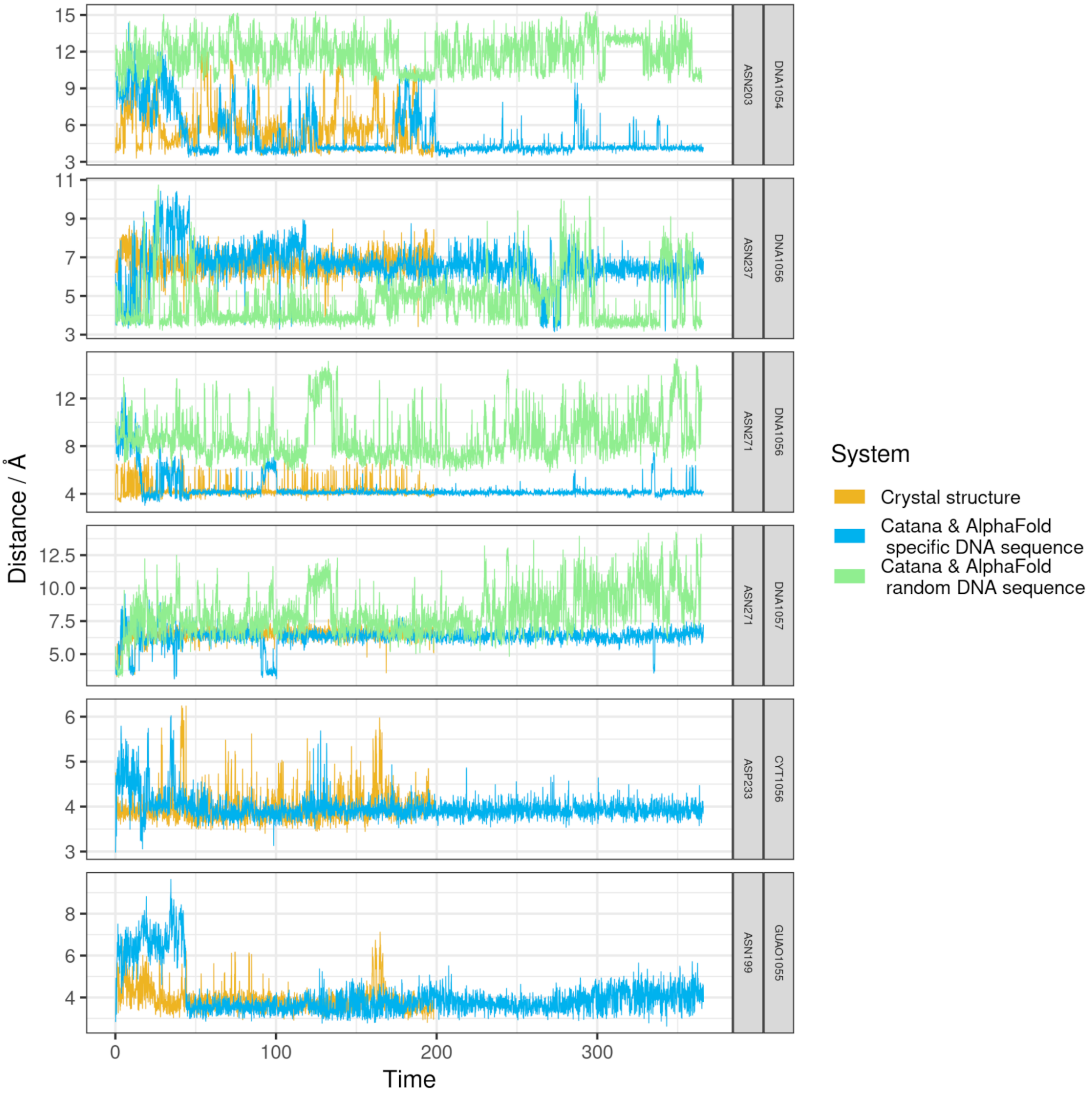

Figure S6. In order to check whether the protein-DNA complex built in Catana adequately reproduced protein-DNA interactions present in the crystal structure, we measured the distance between the key atoms for non-specific protein-DNA backbone phosphate as well as specific protein-DNA base interactions during the MD simulations. After geometry optimisation (SD, 3000 steps) and equilibration with temperature incensement (NVT, followed by NTP), MD simulations were conducted at 310 K using the Langevin thermostat with the gamma value 3.0 (Amber20 using bsc1 force field). An explicit solvent model (TIP3) with PBC was used for MD simulations. The top three plots represent non-specific interactions measured between the carboxyl oxygen of asparagine residues and the DNA phosphate atom. The interaction between DNA residue 1054 and ASN203 is present during the MD simulations of the crystal structure system and the Catana system with the specific sequence, but is absent in the Catana system with a random DNA sequence. Interaction DNA1056-ASN271: the two bottom plots show the distances between asparagine carboxyl atoms and nucleotide bases, which we consider to be specific interactions. There is no distance present for the random sequence system because these nucleotides are not present at the same positions in this system due to a different DNA sequence. The described results show that the specific protein-DNA interactions present in the crystal structure were reproduced during the simulations of the sequence-specific system, but are absented from simulation of the system with a random DNA sequence.

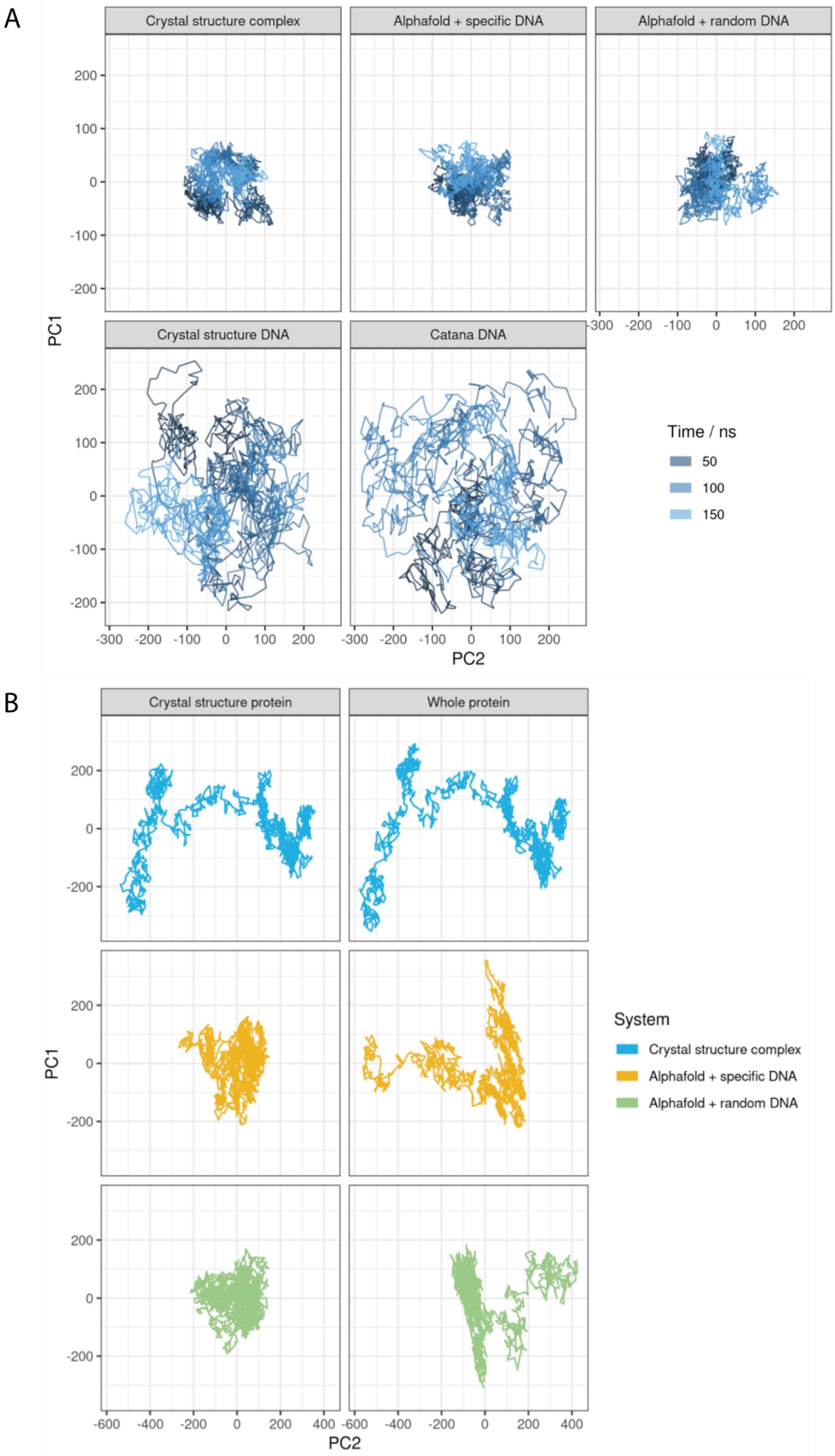

Figure S7. A principal component analysis (PCA) was performed on all non-hydrogen atoms using cpptraj from the Amber20 simulations package for the following systems: (i) the system prepared starting from the crystal structure (PDB ID: 3UGM), (ii) the system was prepared from Catana, with the same DNA sequence as in the crystal structure (system (ii)), and the system with a random DNA sequence (system (iii)). The PCA was performed on the whole protein, on the portion of the protein present in the crystal structure, and also on the DNA molecule itself. For the last analysis, we also included simulations of DNA-only systems. (A) By comparing the size of the PC1 and PC2 space for DNA molecules in a complex with the protein (up) and for free DNA molecules, it can be concluded that the DNA molecules in protein-DNA complexes are dynamically "locked". (B) The size of the PC1 and PC2 space for the crystal structure simulations is somewhat larger than for the other two simulations. More interestingly, while for the systems built in Catana the space is greatly reduced when only the middle portion (residues 68 to 946, crystal structure protein) is taken into account, for the crystal structure simulation this is not the case.

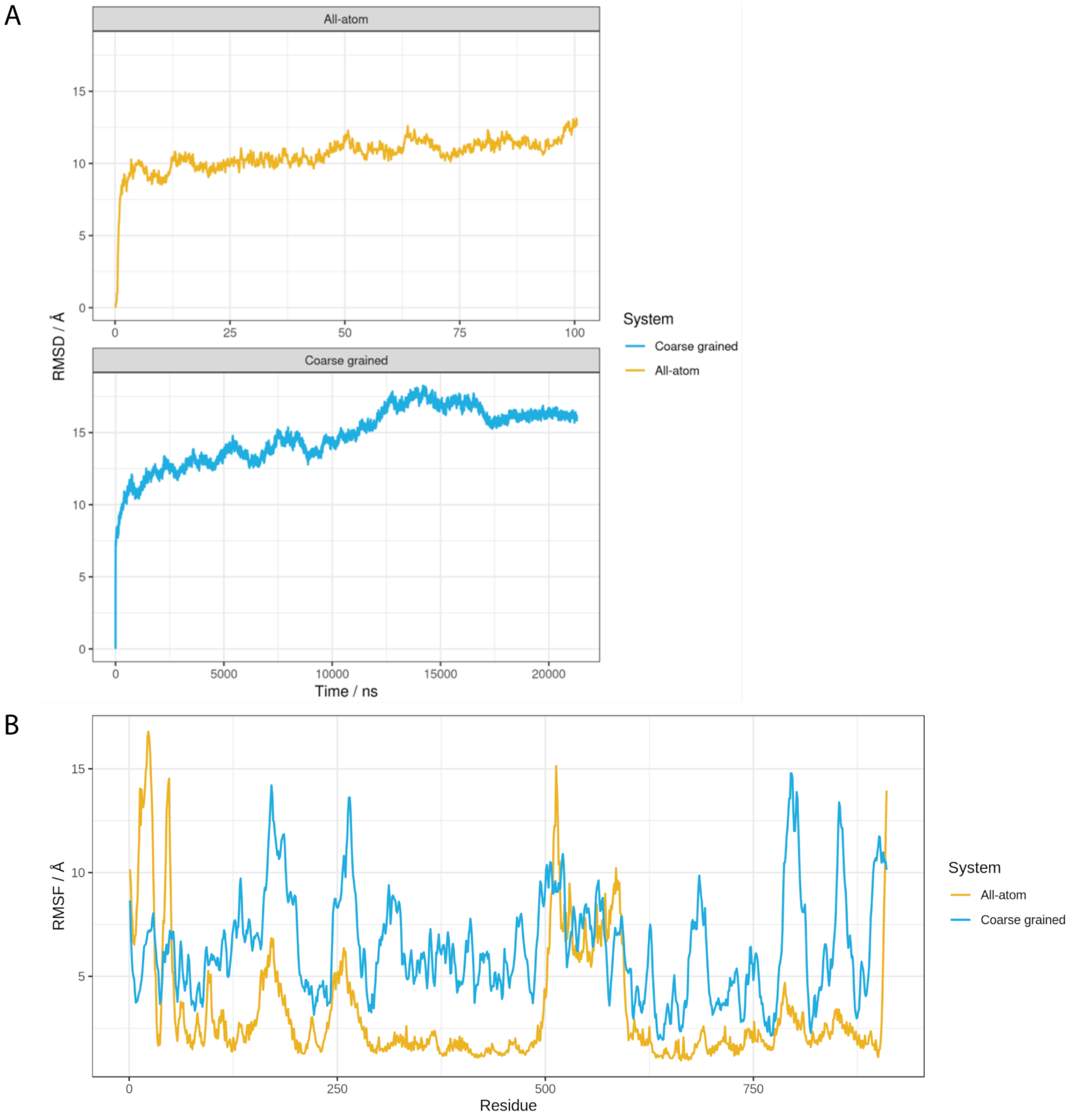

Figure S8. The colicin-HRP fusion proteins were built in Catana using two approaches. (Fig1A) The crystal structures of colicin and HRP were imported from the PDB file and manually connected via linker residues in Catana. (Fig1B) The sequence of the fusion protein was passed to Catana and built using AlphaFold. The system in Fig1B was simulated as described in Figure S4. Additionally, it was subjected to coarse-grained molecular dynamics using the martini22 force field and a timestep of 20 fs. Prior to MD simulations, the system were geometry optimised (SD, 1000 steps), solvated, and equilibrated. (A) The RMSD and (B) RMSF were calculated during the coarse-grained simulations.
